# Cancer Stem Cells and Side Population Cells in Breast Cancer and Metastasis

**DOI:** 10.3390/cancers3022106

**Published:** 2011-04-19

**Authors:** Kelly M. Britton, John A. Kirby, Thomas W.J. Lennard, Annette P. Meeson

**Affiliations:** 1 Institute of Genetic Medicine, Newcastle University, International Centre for Life, Central Parkway, Newcastle-upon-Tyne, NE1 3BZ, UK; E-Mail: k.m.britton@ncl.ac.uk (K.M.B.); 2 Institute of Cellular Medicine, Newcastle University, 3rd Floor William Leech Building, Framlington Place, Newcastle-upon-Tyne, NE2 4HH, UK; E-Mail: j.a.kirby@ncl.ac.uk (J.A.K.); 3 Faculty of Medical Sciences, Newcastle University, 3rd Floor William Leech Building, Framlington Place, Newcastle-upon-Tyne, NE2 4HH, UK; E-Mail: t.w.j.lennard@ncl.ac.uk (T.W.J.L.); 4 North East England Stem Cell Institute, Bioscience Centre, International Centre for Life, Central Parkway, Newcastle-upon-Tyne, NE1 3BZ, UK

**Keywords:** breast cancer, cancer stem cells, side population cells, metastasis

## Abstract

In breast cancer it is never the primary tumour that is fatal; instead it is the development of metastatic disease which is the major cause of cancer related mortality. There is accumulating evidence that suggests that Cancer Stem Cells (CSC) may play a role in breast cancer development and progression. Breast cancer stem cell populations, including side population cells (SP), have been shown to be primitive stem cell-like populations, being long-lived, self-renewing and highly proliferative. SP cells are identified using dual wavelength flow cytometry combined with Hoechst 33342 dye efflux, this ability is due to expression of one or more members of the ABC transporter family. They have increased resistance to chemotherapeutic agents and apoptotic stimuli and have increased migratory potential above that of the bulk tumour cells making them strong candidates for the metastatic spread of breast cancer. Treatment of nearly all cancers usually involves one first-line agent known to be a substrate of an ABC transporter thereby increasing the risk of developing drug resistant tumours. At present there is no marker available to identify SP cells using immunohistochemistry on breast cancer patient samples. If SP cells do play a role in breast cancer progression/Metastatic Breast Cancer (MBC), combining chemotherapy with ABC inhibitors may be able to destroy both the cells making up the bulk tumour and the cancer stem cell population thus preventing the risk of drug resistant disease, recurrence or metastasis.

## 1. Introduction

In breast cancer the primary tumour is never the cause of death; the development of metastatic disease is the major cause of cancer related mortality [1]. In the UK, breast cancer survival rates have improved greatly over the last few decades with a 5-year relative survival rate of 82% between 2001 and 2006 [2]. However this rate is reduced dramatically with the onset of metastatic disease, with a 5-year survival rate of only 12.98% recorded in 2002 for breast cancer patients with stage IV breast cancer [2].

Metastatic breast cancer (MBC) and its causes therefore remains a major concern with approximately 30% of patients with early stage breast cancer eventually progressing to metastatic disease [3]. Currently there is no standardised treatment for MBC. First-line treatment often involves the use of an anthracycline and/or taxane with or without endocrine treatment, and while results with these agents may be initially encouraging, frequently disease progression occurs within 6–10 months [3]. It is thought that the development of chemotherapy resistance can be attributed to treatment failure in over 90% of MBC patients [3]. If progression occurs or the patient is resistant to anthracycline/taxanes they can be offered capecitabine, gemcitabine, vinorelbine or albumin-bound paclitaxel with average response rates of 20–30% [3]. Unfortunately, these second-line agents offer little or no benefit to long term patient outcome [3].

Cancer develops through the progressive accumulation of genetic mutations that transforms normal cells into a malignant phenotype. Carcinogenesis results in cells acquiring more primitive cell characteristics, such as the ability to self-renew and an infinite capacity to divide [4,5], which can occur as a result of mutations affecting self-renewal, cell survival, proliferation and apoptosis [6,7]. There are two main hypotheses on the origin of cancer:

1. Stochastic theory;
2. Hierarchy (Cancer Stem Cell) theory.

The stochastic theory predicts that every cell within a tumour can generate a new tumour when in the correct microenvironment [6]. However, the probability of these cells entering the cell cycle is low [8]. This would suggest that tumours are relatively homogeneous in nature. Evidence to support this theory is that cancer cells within the same tumour tend to express a variation of markers representative of normal cellular differentiation [9]. Studies have also shown that mouse fibroblasts can be induced to form pluripotent stem cells [10] and mature rat hepatocytes can form small hepatocyte-like progenitor cells which can regenerate the liver after retrorsine injury [11]. However, primordial germ cells, which form teratocarcinomas were found to be no more differentiated than there malignant counterparts [4,8]. For example, if cancer is believed to be a result of differentiated cells gaining mutations that increase their life span, these cells will not express stem cell markers. Also, these cells are terminally differentiated, proliferate for a short period of time and are subject to a high rate of turnover, which means that they are unlikely to accumulate enough mutations to undergo neoplastic transformation [12].

The cancer stem cell (CSC) theory has been attributed to Cohnheim (1875) who postulated that stem cells misplaced during embryogenesis may be responsible for tumour formation later in life [13-15]. Histological analysis of embryonic and neoplastic tissue and comparison of their growth characteristics shows many similarities [16], for example they are both able to self-renew, differentiate, resist damaging agents, are long-lived, able to migrate and have extensive proliferative potential [17,18]. Studies on teratocarcinomas show that they are heterogeneous tissues containing a variety of undifferentiated and differentiated cell types which is consistent with tissues derived from stem cells. Pierce (1974) expanded on Cohnheim's theory [15] hypothesising that neoplasms develop from normal stem cells which do not escape developmental influences as suggested by Cohnheim but instead develop and function normally until carcinogenesis alters intercellular controls regulating their proliferation [19]. More recently the CSC model has been referred to as the hierarchy theory which predicts that only a small number of cells are capable of initiating a tumour but can do so at a high frequency. This suggests that tumours are heterogeneous in nature [5,6,17,20-22]. There are several reasons to believe that stem cells may be involved in carcinogenesis, for example there is evidence that most tumours develop over months or years which requires the transformational mutations to occur in cells with a long-life span. The longevity of stem cells means that they could accumulate enough mutations to undergo neoplastic transformation [13,18,22-24]. Self-renewal ability of stem cells also means that it would require fewer mutations to become neoplastic compared to more differentiated cell types [25,26].

Over the last few years there has been a surge in the number of studies investigating the role of CSC in tumour progression, however most research has focussed on the involvement in the primary tumour. CSC are tumour cells that are capable of self-renewal and give rise to heterogeneous tumour masses [13,27,28]. They are defined as ‘cells that have the ability to form tumours in immunodeficient mice that recapitulate the heterogeneity of the tumour from which they were isolated [27]’, and thus they share many properties with stem cells, being long-lived, self-renewing and highly proliferative. They have increased resistance to chemotherapeutic agents and apoptotic stimuli and have increased migratory potential above that of the bulk of tumour cells [29-34]. The origin of CSC remains unclear. It is unknown if CSC are dedifferentiated cells that have acquired a more stem cell-like phenotype or if they are stem cells which through longevity have accumulated the sufficient number of mutational hits required for carcinogenic transformation to occur or if they are due to a combination of both these factors. Evidence suggests that conventional chemotherapy targets the bulk of the tumour cells allowing slow cycling cells such as CSC to persist after treatment and promote further/metastatic disease [13].

## 2. Metastasis

In 1889, Paget devised the “seed and soil hypothesis” of cancer metastasis in which he suggested that the seeds of cancer (the tumour cells) have an ability to survive in specific soils (organs) that are conducive to their growth [35]. This hypothesis could easily refer to the properties of CSC. The metastatic process is complex, non-random and involves a long series of interrelated steps, any of which may be rate-limiting [36-39]. Metastatic steps include: progressive growth of the primary tumour which requires the establishment of a good blood supply through angiogenesis, in which growth factors regulate the breakdown of the extracellular matrix (ECM) and also cause the proliferation and migration of endothelial cells [37,40]. Cells capable of metastasis also acquire the ability to invade [37]. For epithelial cancers this involves cells undergoing an Epithelial-Mesenchymal Transition (EMT). EMT involves epithelial cells losing their epithelial characteristics and acquiring a more mesenchymal phenotype which occurs as a result of cytoskeletal changes within the cells. These changes allow the cell to acquire a more migratory phenotype [36,41], increasing the probability of tumour cells entering the blood and lymphatic systems. This process is influenced by chemokines and their receptors which are thought to play an important role in tumour development by influencing tumour transformation, survival, proliferation, invasion and metastasis and also regulation of angiogenesis and tumour-leukocyte interactions. Despite this, the majority of circulating tumour cells appear to be destroyed [37]. Those that persist may acquire the ability to metastasise and once inside the target organ may undergo Mesenchymal-Epithelial Transition (MET), proliferate and if the environment is conducive the disseminated cells may grow to establish a new tumour thus completing the metastatic process [38].

## 3. Role of Breast Cancer Stem Cells in Metastasis

There is accumulating evidence which suggests that CSC may play a role in metastasis [42]. Breast cancer stem cells can be identified using several methods [43]:

1. Mammosphere—Forming ability [44-46];
2. Flow cytometry—Analysis of breast cancer stem cell markers including CD44 [17], CD24 [47,48], CD49f [49-51] and aldehyde dehydrogenase 1 (ALDH1) [52]. Cells are then sorted and characterised further using clonogenicity, proliferation, differentiation and *in vivo* tumourigenicity assays;
3. Label retention studies—For example radioactive thymidine and BrdU [53-55];
4. Quiescence—Pece and colleagues (2010) have isolated normal human mammary stem cells using the lipophilic dye, PKH26 which is retained by quiescent cells [56];
5. Functional assays—Side Population (SP) cells which have an increased ability to efflux Hoechst 33342 dye [57] and ALDH-positive cells that are identified using the Aldefluor assay which identifies cells with high aldehyde dehydrogenase activity [52,58];

The use of cell surface markers to identify CSC from solid tumours requires careful optimisation of antibodies and the gated populations must be validated using both *in vitro* and *in vivo* functional assays. Discrepancies in marker expression may arise due to the manipulations required to prepare the samples for analysis, for example tumour dissociation into single cell suspensions and culturing of cells may alter cell behaviour and viability. Unlike normal tissue stem cells, CSC are influenced by the specific genetic aberrations of the tumour, by the stage of the disease and also by any therapeutic interventions given to the patient [59]. In patient tumours, CSCs are ‘moving targets’ (cells that are continually evolving) making it difficult to isolate these cells in the clinic. The clinician needs to be able to identify the different CSC populations in the patient throughout all stages of disease progression and needs to be able to target this population without detrimental effects on the normal stem cell populations [27,59].

At present most of what we know about the role of CSC in breast cancer metastasis has been based on ALDH-positive, CD133-positive or CD44^+^CD24^−/low^ breast CSC populations [58,60-62]. Breast cancer is a heterogeneous disease which can be classified into different subtypes based on histology or molecular profiling [63,64]. It is becoming apparent that different breast cancer subtypes may result from different breast cancer cell populations. ALDH has been used to identify hematopoietic stem cells (HSC) in both mice and humans [52,65-67], normal mammary stem cells, and also CSC populations in AML [68], multiple myeloma [67,69], and malignant human mammary epithelial cell lines [52]. In xenograft human breast tumours, these cell types displayed cancer stem cell properties giving rise to tumours *in vivo* that recapitulated the heterogeneity of the tumour from which they were isolated [52]. ALDH^+^ cells have been shown to mediate invasion and metastasis in inflammatory breast cancer [70] and this population is increased in basal breast cancer cell lines and in breast tumours from patients following neoadjuvant chemotherapy [71]. In fact, ALDH1 has been found to be an independent predictive factor for early metastasis and decreased survival in patients [70]. Marcato and co-workers assessed the expression of 19 ALDH isoforms and demonstrated that CSC in breast tumours could be identified using the ALDH1A3 isoform and it was shown that the expression of ALDH1A3 correlated with breast tumour grade and stage and correlated prevalence of CSC with metastatic breast cancer [72].

Independently, both CD44^+^ [73,74] and CD24^+^ [47,75] breast cancer cell populations have been shown to be involved in the metastatic process. Data on the role of CD44^+^ or CD24^−^ cells in metastatic tissues is conflicting, Shipitsin and co-workers determined that the CD24^+^ cell population was increased in distant metastases regardless of the site of metastases [74]. However, no correlation was observed when comparing CD44^+^ cells in primary invasive tumours and matched samples taken from metastatic sites [74]. CD44 is subject to extensive alternative splicing and different splice variants of CD44 have been shown to be involved in metastasis in a variety of different cancers and have been associated with poor patient outcome [76]. For example CD44v3, CD44v6 and CD44v7/8 variants have been shown to correlate with more aggressive breast cancer subtypes [76]. Brown and colleagues have demonstrated that it is essential for a switch in CD44 expression from CD44 variants (CD44v) to CD44 standard isoform (CD44s) for Epithelial-Mesenchymal Transition (EMT) to occur *in vivo* [77]. In 2003, Al-Hajj and colleagues identified a population of cells in human breast cancers with a Lin^−^ESA^+^CD44^+^CD24^−/low^ phenotype that were highly tumourigenic *in vivo* and were the only cell population capable of forming tumours in NOD/SCID mice that recapitulated the heterogeneity of the tumour from which they were isolated [17]. Sheridan and co-workers (2006) discovered CD44^+^CD24^−/low^ populations constituted a high proportion of the total cell population in basal or myoepithelial breast cancer cell lines [47]. These cell lines expressed high levels of the proinvasive genes Il-1α, Il-6 and Il-8 and were more invasive than cell lines containing less than 30% CD44^+^CD24^−/low^ cells [47]. Subsequently, studies have shown that CD44^+^CD24^−/low^ cells are slow cycling, chemoresistant, capable of forming mammospheres and are enriched in drug resistant cell lines [78,79].

Studies correlating the presence of CD44^+^CD24^−/low^ cells in breast tumours with patient follow up have produced conflicting data. Abraham and colleagues determined that 78% of breast cancer cases assessed contained a CD44^+^CD24^−/low^ population constituting less than 10% of the total cell population [80]. No significant correlation between the prevalence of CD44^+^CD24^−/low^ expressing cells and response to different therapeutic regimes was observed [80]. Conversely, Li and co-workers showed that the proportion of cells with the CD44^+^CD24^−/low^ phenotype was significantly increased following neoadjuvant chemotherapy [81]. The majority of studies investigating CD44^+^CD24^−/low^ cells have utilised antibodies that do not discriminate between CD44 isoforms. In the future it would be beneficial to investigate expression of specific CD44 isoforms and their affect on the CD44^+^CD24^−/low^ phenotype.

In breast cancer, CD133 appears to play less of a role as a stem cell marker than in other cancers [61]. However, CD133^+^ cells have been identified in breast cancer cell lines created from Brca1-deficient murine mammary tumours and these cells were highly tumourigenic, expressed common stem cell markers and were resistant to DNA-damaging chemotherapy [79]. A population of cells with a CD44^+^CD29^hi^CD133/2^hi^ phenotype has also been identified in ERα-negative human breast tumours that was highly tumourigenic, capable of self-renewal and also expressed common stem cell markers [82].

## 4. Side Population Cells

Another putative CSC population are Side population (SP) cells. SP cells are identified using dual wavelength flow cytometry combined with Hoechst 33342 dye efflux [57,83]. SP cells have an elevated rate of Hoechst efflux which is attributed to the expression of one or more members of the ABC transporter family [34,84]. The SP phenotype is a functional ability within this cell population and is not reliant on the use of cell surface markers. Many groups are assessing the role of SP cells in different cancers however, results need to be analysed cautiously. Hoechst 33342 is a DNA binding dye which is toxic to cells at high concentrations and this is exacerbated by exposure to UV light. It is important to optimize the Hoechst 33342 dye concentration, the incubation time and cell counting in order to minimise cell toxicity [85,86]. Some studies have shown that Hoechst 33342 dye can impair cell differentiation [87], clonogenicity [88,89] and tumourigenicity [87,90-92]. Cell viability may also be affected by Hoechst 33342 dye [88] or by the cell sorting process [89,90] and therefore it is important to perform a functional assessment of cell viability to overcome this issue [86].

SP cells were first identified in murine bone marrow and were thought to be a stem cell (a cell type that has the ability to self-renew and differentiate to give rise to more restricted cell types [13]) population as they expressed the phenotypic markers of multipotent Haematopoietic Stem Cell (HSC; Lin^−^SCA-1^+^), were capable of contributing to both the lymphoid and myeloid cell lineages and were highly enriched in HSC activity [57]. SP cells have since been identified in a diverse array of normal tissues including, lung, heart, endometrium and mammary gland [93-96]. Several cancer cell lines have also been shown to contain an SP population including breast cancer, lung cancer, prostate cancer and ovarian cancer cell lines [20,97-99]. SP cells have also been identified in solid tumours including mesenchymal tumours, breast tumours, neuroblastomas and ascites extracted from ovarian cancer patients [20,91,99-101]. SP have been shown to possess stem cell-like properties within these tissues [20,91,93-100]. However in some studies, SP cells have not been shown to have stem-cell properties. CSC-like cells have been shown to play an important role in glioblastoma multiforme (GBM) [102]. Broadley and colleagues found that neurosphere formation enriched for stem cell-like activity [102]. GBM SP cells were not enriched in neurospheres, were unable to self-renew and had a lower tumourigenic potential than non-SP (NSP) cells [102]. SP cells in adrenocortical tumours did not have increased ability to proliferate or self-renew and were not more chemoresistant than the NSP population [103]. In 2006, Shackleton and co-workers demonstrated that a single cell with a Lin^−^ CD29^hi^CD24^+^ phenotype could function as a mammary gland stem cell reconstituting an entire functional murine mammary gland [48]. SP cells were also identified in the murine mammary gland but they did not identify the Lin^−^CD29^hi^CD24^+^ population [48].

In haematopoietic cells, the SP phenotype has been defined by the expression of ABCG2 [34,84], an ABC half-transporter involved in xenobiotic protection [104] and commonly involved in multidrug resistance [105]. The importance of ABCG2 expression on the SP phenotype is demonstrated by studies on Abcg2 null mice which have been shown to have reduced numbers or fewer detectable SP cells than wild-type mice. Haematopoietic cells from Abcg2^−/−^ null mice were shown to be more sensitive to mitoxantrone, a drug commonly used in chemotherapy [84]. Incorporating the green fluorescent protein (GFP) reporter gene into the Abcg2 locus in mice showed that 91% of haematopoietic SP cells were found to express the Abcg2/GFP allele supporting the role of ABCG2 in the SP phenotype [106].

## 5. Characterisation of SP in the Normal Mammary Gland

SP cells have been identified in both mouse and human mammary gland tissue [93,107]. Analysis of the murine mammary gland of Abcg2/Abcb1a/1b triple knockout mice showed almost complete loss of mammary gland SP cells, demonstrating in the murine mammary gland that both ABCG2 and ABCB1 contribute to the mammary gland SP phenotype [108]. It is interesting to note that higher percentages of SP cells have been identified in hyperplastic mammary glands from MMTV-Wnt-1 and MMTV-ΔN-catenin murine models compared to the wild-type control [109]. This suggests that the percentage of SP cells increases during breast cancer development and progression in some breast cancer subtypes.

At present there is a debate whether mammary gland SP cells are stem cells or a more-restricted mammary gland progenitor cell population. However, regardless of their status, studies have shown that human mammary gland SP cells are able to generate luminal and myoepithelial lineages [93], to form branching structures in matrigel and preferentially form spheres *in vitro* [110,111].

## 6. SP cells: A Presence in Numerous Cancers and Cancer Cell Lines

Patrawala and colleagues (2005) found that 30% of human cancer cells and xenograft tumours contain SP cells [98]. Extensive studies have attempted to characterise SP cells isolated from cancer cell lines and have identified shared properties between cancer SP cells. SP cells isolated from cancer cell lines have been found to be capable of self-renewal [99], can undergo asymmetric division [20,31,97-99,112,113] and express common stem cell markers including Notch1 and Bmi1 [20,97,98,114]. In the majority of studies, SP have also been found to be chemoresistant [20,97,99,112,115], radioresistant [114,116] and have increased invasive potential *in vitro* [117] compared to NSP cells [97,112,115]. *In vivo* studies have also demonstrated that in most cancer cell lines SP are more tumourigenic [97,99,113-115,118,119] and have greater metastatic potential [118,120,121] than their NSP counterparts [122].

## 7. SP in Breast Cancer Cell Lines

Extensive work has been performed to characterise SP cells in breast cancer cell lines [20,92,98,123-126]. Purified SP from the MCF7 breast carcinoma cell line had increased expression of the ‘stemness genes’ Notch1 and β-catenin [98], have a large nuclear-to-cytoplasmic ratio compared to NSP cells (a stem cell characteristic) and breast cancer cell line SP populations are capable of asymmetric division *in vitro*. In contrast, NSP cells could only generate NSP cells *in vitro* [92,113,123]. *In vivo*, breast cancer SP cells have been shown to be more tumourigenic than NSP cells when transplanted into immunodeficient mice [92,98,113,121].

The importance of ABC transporters in defining the SP phenotype in normal tissues has led to an abundance of studies investigating the role of these transporters in breast cancer SP cells. Many studies have reported increased transcriptional expression of ABCG2 in SP compared to NSP cells [92,98,121,123] in several breast cancer cell lines, and this has been confirmed at the protein level [113]. Targeting ABCG2 with ABCG2 inhibitors or siRNA has been shown to eliminate the SP population in the MCF7 cell line [113]. Studies have determined that MCF7 SP are more radioresistant [116] and chemoresistant to agents including mitoxantrone and carboplatin [92,113,121] than the NSP cell population. MCF7 SP cells have also been shown to have a CD44+CD24^−^CK18^+^EpCAM^+^CK19+ phenotype [121,124] and both MCF7 SP and CD44^+^CD24^−/low^ cells are enriched in mammosphere formation and both populations are preferentially inhibited by NF-κβ pathway inhibitors compared to their non-stem cell counterparts [126]. These properties would suggest that SP cells may play an important role in breast cancer progression in some breast cancer subtypes. Perhaps the presence of an SP population may be indicative of a cohort of patients within a breast cancer subtype that have a poorer response to particular therapeutic options and thus these individuals may have a poorer prognosis. Conversely, in other breast cancer subtypes where SP are not prevalent another breast cancer stem cell population may be responsible for poor prognosis/chemotherapeutic resistance.

There is evidence to suggest that SP cells may undergo EMT and numerous studies have demonstrated an association between EMT, chemoresistance and stem cells [41,44,127,128]. Inducing EMT in the breast cancer cell line MCF7 by the addition of EGFR has been correlated with increased invasive potential and an increased resistance to tamoxifen [129]. Also, doxorubicin induced EMT in murine 4T1 breast cancer cells has been shown to lead to an enrichment of Sca-1-positive cells with an increased invasive potential [130]. SP cells have been shown to be more responsive to TGFβ signalling than NSP cells; with TGFβ treatment increasing the invasive potential and inducing EMT in the SP population in pancreatic cancer cell lines [31]. Upon removal of TGFβ treatment, pancreatic cancer SP cells were also capable of MET [31].

Cancer SP cells are known to be chemoresistant and this has been attributed to the expression of ABC transporters [20,99,131]. Unlike the study on pancreatic cancer SP cells [31], it has been shown that in the MCF7 breast cancer cell line, TGFβ induced EMT and depleted the SP population, down-regulated ABCG2 gene expression and increased cell sensitivity to mitoxantrone [113]. The removal of TGFβ restored the SP phenotype and ABCG2 expression [113]. Knockdown of e-cadherin reduced the SP population but did not affect expression of ABCG2 mRNA or protein thus providing evidence that the EMT response reflects post-translational regulation of ABCG2 function by e-cadherin as well as transcriptional repression of the ABCG2 gene [113]. These studies need to be repeated using other cancer cell lines and primary tumour SP cells in order to have a better understanding of the involvement of SP in EMT.

## 8. SP in Breast Tumours

SP cells have been identified in a diverse array of breast cancer cell lines that are classified within different breast cancer subtypes [123]. However, Clarke and co-workers (2005) have identified SP cells in normal human breast tissue and showed that these cells were ER-positive [111]. This has resulted in it being postulated that SP cells may be predominantly identified in luminal breast tumours [132]. Analysis of putative breast cancer stem cell markers in primary breast cancers demonstrated that different breast cancer stem cell populations were prevalent in different breast cancer subtypes. Basal-like tumours were found to contain a higher proportion of cells with a CD44^+^CD24^−/low^ [47] or ALDH1^+^ [52] phenotype whilst Her2-positive cancers predominantly contained ALDH1^+^ cells. This study did not identify a prevalent breast cancer stem cell population in luminal breast tumours.

To date only Nakanishi and colleagues have identified SP cells in clinical breast tumours which were characterised as luminal cancers [100]. The percentage of SP cells was higher in luminal type (predominantly ER^+^PR^+^Her2^+^) breast cancer cell lines and these cell lines had increased colony forming ability *in vitro* compared to basal a (CK5^+^ and CK14^+^) or basal b (vimentin^+^) subtypes. A strong correlation was observed between Her2 expression and SP presence with the percentage of SP increasing in cell lines enforced with expression of Her2. Treatment with the Her2 signalling inhibitors, tyrophostin AG825 or trastuzumab, decreased the proportion of SP cells in breast cancer cell lines and also inhibited tumour growth *in vivo* [100]. The authors concluded that their data highlights the important role that Her2 plays in regulating SP cells and could account for the poor chemotherapeutic response and aggressive nature of Her2-positive breast cancers [100]. Unfortunately the media used in this study selected for primary breast cancer cells that had a luminal nature which means as yet it is unknown if SP cells can be identified in other breast cancer subtypes. This highlights the importance of identifying SP cells in all patient breast tumour subtypes and relating the presence of these cells to patient follow up in order to elucidate the role of SP cells in breast cancer and potentially to highlight new therapeutic targets. It may also be beneficial to use the identification of patient SP cells as a tool to determine which patients require a more aggressive treatment regime due to increased expression of MDR transporters that may be specifically tailored to eradicate this population of cells.

## 9. SP and Breast Cancer Metastasis

As yet there is no published literature investigating the metastatic potential of SP cells in breast cancer. However, studies in other cancers have shown that SP cells may play a role in metastatic spread of disease [95,120]. Kato and co-workers injected SP and NSP flow cytometrically sorted cells from the Hec-1, human endometrial cancer cell line, subcutaneously into immunodeficient mice and discovered that tumours derived from SP cells were highly invasive with cells invading through the basement membrane into the spine, peritoneum, leg and bone [95]. In contrast, NSP cell derived tumours were encapsulated and clearly separated from the basement membrane of the mouse skin [95]. SP isolated from the pancreatic cancer cell line PANC-1 have been shown to have an increased invasive potential *in vitro* and increased metastatic potential *in vivo* when compared to NSP cells using a murine liver metastasis model [31]. Nishii and colleagues (2009) have shown SP from gastric cancer cell lines have increased expression of several adhesion molecules which resulted in SP having a greater potential to form peritoneal metastases [120] and the prevalence of SP cells has been shown to significantly increase in hepatocellular carcinoma cell lines in a stepwise manner dependent on the cell lines metastatic potential, with the highest percentages observed in the most metastatic cell line [131]. In primary mesenchymal neoplasms, the percentage of SP cells has been shown to correlate with the tumours grade [91]. However to date the presence/percentage of SP cells in primary neoplasms has not been correlated to metastasis.

## 10. CXCR4, SP cells and Breast Cancer

The role of the CXCR4/CXCL12 axis in breast cancer metastasis has been well documented [133-137]. Several studies have shown how acquisition of the chemokine receptor CXCR4 by breast cancer cells can promote specific, chemotactic recruitment to remote sites such as the bone marrow, lymph nodes, liver and lung in response to localised expression of the specific chemokine ligand, CXCL12 [133]. These studies tie in with the distinct pattern of tumour dissemination to regional lymph nodes, lung, liver and bone marrow known to occur as part of the metastatic spread of breast cancer [133]. Stromal cells in all of these sites have been shown to highly express CXCL12 and were able to induce chemotaxis and invasion of breast cancer cells *in vitro* [133]. Several studies using gene silencing strategies to reduce CXCR4 expression have demonstrated that this results in impaired invasion of breast cancer cells *in vitro* and inhibits breast cancer metastasis *in vivo* [133-136]. Primary human breast cancer cells can also respond to CXCL12 [133] and in breast cancer patients, high levels of CXCR4 expression have been correlated with lymph node metastasis in invasive ductal carcinoma and associated with poor overall survival [137,138].

## 11. SP and CXCR4

SP cells are known to be capable of migration in response to CXCL12 [139,140]. Cardiac SP cells transplanted into non-infarct myocardium were shown to be capable of migrating into damaged myocardial tissue. This migration *in vitro* and *in vivo* being due to the interaction between CXCR4 and CXCL12 [140]. Human bone-marrow derived SP cells have also been shown to be CXCR4-positive and capable of migrating in response to CXCL12 *in vitro* [139]. Perhaps as in the case of pancreatic cancer (where the CD133^+^CXCR4^+^ cell population is responsible for metastasis [30]), it is the CXCR4-positive SP population of breast cancer cells that are responsible for breast cancer metastasis.

## 12. CSC in Primary and Metastatic Breast Disease

Current treatment for MBC is limited and has little clinical success [3]. There is a high rate of disease progression in MBC patients who initially respond to treatment with over 90% of MBC patients developing drug resistant disease [3]. If the SP phenotype in breast cancer is defined by the presence of ABC transporters this would make them an ideal candidate for contributing to drug resistant MBC. If metastases are seeded from SP cells this would result in an abundance of MDR-expressing cells making the tumours highly resistant to therapeutic intervention and therefore extremely difficult to eradicate [141].

To clarify the role of SP cells in MBC, the identification of SP cells needs to be performed in all breast cancer subtypes and their presence related to the tumour characteristics and to patient follow up. Breast cancer SP cells need to be isolated from patients and extensively characterised which will involve the need to transplant these human cells into animal cancer models to elucidate the potential of these cells to contribute to invasion and metastasis. A manuscript published by Michael Clarke's group described the development of a human-in-mouse breast cancer xenograft model in which the metastatic potential of breast cancer stem cells could be determined [142]. This group labelled breast cancer stem cells with Luc2-eGFP or Luc2-Tom enabling as few as 10 labelled cells to be visualised *in vivo* and *ex vivo* [142]. It will be necessary to obtain lymph node and primary tumour samples from patients undergoing surgery for breast cancer and to determine the presence of SP within these samples. If SP are indeed present in lymph nodes of breast cancer patients it could be indicative of patients likely to progress with distant metastatic disease. It is already known that the presence of metastases in axillary lymph nodes is associated with more aggressive breast cancers and poor disease-free and overall survival in breast cancer patients [143,144]. A disparate hormone receptor status between primary breast tumour tissue and nodal tissue has been observed demonstrating the importance of assessing breast cancer stem cells in both primary tumour and lymph node samples [145]. Lu *et al.* (2010) detected higher proportions of CD44-positive cells in primary tumour samples analysed from lymph node positive supraglottic carcinoma patients compared to lymph node negative patients thus showing the importance of identifying CSC in lymph node samples [146].

## 13. Treatment Strategies to Target Cancer Stem Cells, Including SP Cells

The emerging role of cancer stem cells in breast cancer development and progression has led to intense work to identify drugs that target the CSC population of drug resistant cells [60]. The importance of finding new therapies to target breast cancer stem cells is highlighted by accumulating evidence showing the resistance of cancer stem cells to chemotherapy and radiotherapy. Transcriptional profiling has been used to define several breast cancer subtypes that require different therapeutic regimes [147]. The heterogeneity of breast cancer stem cell populations in different breast cancer subtypes suggests that several targeted therapies may be necessary. It is hoped that increasing our understanding of the molecular biology of cancer stem cells may identify new therapeutic targets. For example, metaplastic breast cancers have been found to display a similar ‘tumourigenic signature’ to that of the CD44^+^CD24^−/low^ breast cancer stem cell population and MBC have been shown to have aberrant MAPK components which potentially could benefit from treatment with MAPK pathway inhibitors [147].

The majority of studies have focused on targeting the CD44^+^ and CD44^+^CD24^−/low^ cell populations, culminating most recently in the use of Salinomycin, a highly selective potassium ionophore, as a compound that could selectively eliminate the CD44^+^CD24^−/low^ breast cancer stem cell population compared to treatment with paclitaxel alone [148]. *In vivo*, Salinomycin was able to inhibit the formation of tumours in the 4T1 murine breast cancer cell model and also in the ras-transformed HMLE model. Salinomycin treatment also prevented metastasis of 4T1 cells *in vivo* [148,149]. The mechanism of action of Salinomycin is the induction of a distinct apoptotic pathway that does not lead to cell cycle arrest and in this way results in massive apoptosis in cancer cells [117]. Fuchs and colleagues demonstrated that Salinomycin could even activate apoptosis in cells that were chemotherapy resistant or resistant to apoptosis due to the overexpression of factors such as bcl-2, MDR1 or 26S proteasomes with increased proteolytic activity [117]. Perhaps the effect of salinomycin on cells overexpressing MDR transporters would mean that it could be used to specifically target breast cancer SP cells.

As the SP cell phenotype is a consequence of the expression of ABC transporters, strategies to inhibit these might be worth revisiting. Nearly all cancer chemotherapy involves a first-line agent known to be a substrate of an ABC transporter [150], thereby increasing the risk of developing drug resistant tumours. If SP cells do play a role in breast cancer progression/MBC, combining chemotherapy with ABC inhibitors may be able to destroy both the cells making up the bulk tumour and the cancer stem cell population thus preventing the risk of drug resistant disease, recurrence or metastasis [104,141,150]. Results from clinical trials of ABC inhibitors have been disappointing with first line ABC transporter inhibitors for example verapamil being too toxic for use in cancer patients. Second line agents such as Zosuquidar were more potent and less toxic but demonstrated no additional clinical benefit in locally recurrent breast cancer or in MBC patients [151]. However, some of these trials have shown limited benefits, for example, clinical trials with PSC 833 showed that it was necessary to reduce the dose of the chemotherapy used compared to conventional chemotherapy, as combining chemotherapy with PSC 833 resulted in a pharmacokinetic interaction that altered the metabolism of the chemotherapeutic agent [150], thus achieving a statistically significant improvement in relapse-free and overall survival [152]. The trial combining PSC 833 with mitoxantrone, etoposide and cytosine arabinoside in AML patients had to terminate early due to toxicity issues [153]. However, analysis did show a statistically significant improvement in the complete remission rate of patients and a trend towards an improved disease-free survival within the treatment arm of the study [154]. However, the use of a broad-spectrum ABC transporter inhibitor will be unlikely as the 3 main transporters, ABCB1, ABCC1 and ABCG2, involved in multidrug resistance are from three evolutionary and structurally distinct ABC transporter families making it difficult to identify a common target [155].

In addition to chemoresistance, ABC transporters also play a role in cell migration, differentiation and protection against apoptosis [156-158]. In MCF7 breast cancer cells, siRNA knockdown of ABCB1 has been demonstrated to decrease migration and invasion *in vitro* [159]. ABCC1 has also been seen to be more highly expressed in metastatic lymph nodes compared to the primary tumour of patients with metastatic breast cancer [159]. Therefore gene silencing strategies alone or in combination with the use of chemotherapeutic reagents or anti-CSC drugs might prove more effective at targeting CSC in MBC. However, it will be important to determine if the breast cancer stem cell population can be targeted without detrimental effects on the normal stem cell populations.

**Figure 1.**
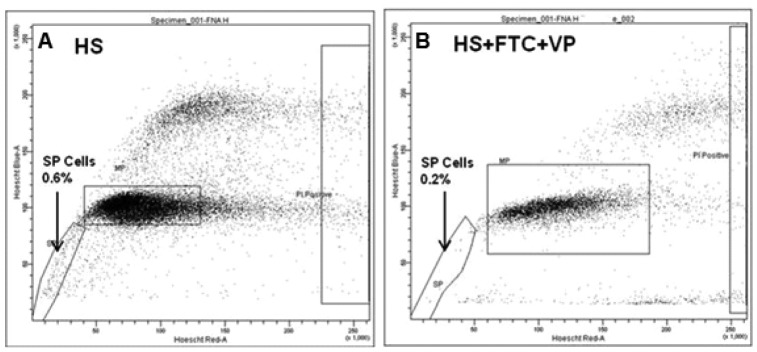
SP profile for a fine needle aspirate taken from a male breast cancer patient. (**A**). A SP population of 0.6% of the total cell population was observed when cells were incubated with 2.5 μg/mL Hoechst 33342 dye; (**B**). The efflux of Hoechst dye by the SP population was partially inhibited by addition of a combination of 10 μM FTC and 50 μM verapamil prior to the Hoechst incubation. * Prior to Hoechst staining samples were lysed twice with Red Blood Cells (RBC) lysis buffer and propidium iodide was added prior to flow cytometry analysis to exclude non-viable cells.

Nakanishi and co-workers (2010) identified SP in primary cells derived from patient breast tumours [100] and we confirm the presence of SP in patient breast tumours through detection of SP cells in Fine Needle Aspirate (FNA) samples from palpable breast tumours. Targeting SP cells may only be relevant to some breast cancer subtypes, such as luminal tumours [100], whilst other therapies may be required for other subtypes shown to be driven by different breast cancer stem cell populations. SP cells have been observed in both female [100] and male breast cancer patients (Meeson laboratory data, Figure 1). This highlights the presence of CSC in male breast cancer, and raises the question can we target CSC in both female and male breast cancer cases using the same strategies? No studies looking at the role of CSC in male breast cancer have as yet been reported.

The importance of Her2 in the regulation of breast cancer SP cells has been demonstrated by Nakanishi and colleagues (2010) and SP cells have been observed to be more prevalent in Her2 overexpressing breast cancer cell lines and luminal breast tumours [100]. The Her2 gene is amplified in approximately 20% of all breast cancer cases and is associated with a more aggressive disease and commonly involves early onset of metastases [29,160]. In fact, breast cancer patients with Her2-positive breast cancer cells in their bone marrow at the time of diagnosis have an increased risk of metastatic relapse and overall have a poorer prognosis [29].

Trastuzumab and Lapatinib are targeted therapies against the Her2 receptor and the Her2 pathway and are usually only prescribed to patients with Her2-positive breast tumours [161] and clinical studies have determined the benefit of giving patients neoadjuvant trastuzumab or lapatinib in both Her2-positive and Her2-negative patients [162,163]. Her2 overexpressing cell lines have increased mammosphere forming efficiency (MSFE) and an increased ALDH^+^ cell population [164]. Treatment of Her2 overexpressing cell lines with Trastuzumab reduced the number of ALDH-positive cells and this subsequently resulted in decreased invasive and tumourigenic potential of these cells [164,165]. Perhaps the benefit of Her2 inhibitors in both Her2-positive and Her2-negative tumours could be due to the interaction of these therapeutic agents with the CSC population or SP cells within these patient tumours.

## 14. Conclusion

Reported *in vitro* studies show promising results for therapeutically targeting breast CSC but they need to be translated into the clinic [117,148,149]. This may be difficult to achieve without standardised methods of detecting and characterising these cell populations both before and after therapy. The knowledge of the particular cancer stem cell properties involved in different breast cancer subtypes may identify new therapeutic targets especially for metastatic breast cancers in which treatment options are currently limited [3,147]. It is important that the role of breast cancer stem cells in metastasis is determined. If cancer stem cells could be targeted in breast cancer therapy the analysis of distant metastases for the presence of cancer stem cell populations may be beneficial in the selection of therapy.

If SP is involved in the metastatic process, therapies to target SP cells will need to be sought in order to minimise toxicity effects often experienced by treatment with systemic therapies. If ABC inhibitors/modulators are to be of use they need to be highly specific in order to reduce systemic effects as ABC transporters play a pivotal role in the blood brain barrier, many tissues and most normal stem cells [155], and interfering with this process could have catastrophic outcomes in patients. Treatments will have to be carefully tested not only on breast cancer SP cells but also on normal SP/stem cell populations to determine the impact they may have on normal body processes [166]. Therapeutic agents will also need to be tested in combination with commonly used breast cancer therapeutics as the effect of each agent alone may be very different to that observed when used in combination.
